# Safety evaluation of calcium L-methylfolate

**DOI:** 10.1016/j.toxrep.2019.09.012

**Published:** 2019-09-26

**Authors:** K.E. Niederberger, I. Dahms, T.H. Broschard, R. Boehni, R. Moser

**Affiliations:** a Leveret GmbH, Aberenrain 30, 6340, Baar, Switzerland; b DSM Nutritional Products, Wurmisweg 576, 4303, Kaiseraugst, Switzerland; c Merck KGaA, Frankfurter Strasse 250, 64293, Darmstadt, Germany; d Merck & Cie, Im Laternenacker 5, 8200, Schaffhausen, Switzerland

**Keywords:** ANOVA, analysis of variance, BaP, benzo[a]pyrene, bw, body weight, EFSA, European Food Safety Authority, GD, gestation day, GLP, Good Laboratory Practice, GRAS, generally recognized as safe, HPLC, High Performance Liquid Chromatography, JECFA, Joint FAO/WHO Expert Committee on Food Additives, L-5-MTHF-Ca, calcium L-methylfolate, 5-MTHF, 5-methyltetrahydrofolate, MTT, 3-[45-dimethylthiazole-2-yl]-2,5-diphenylbromide, NNG, net grains/nucleus, NOAEL, No Observed Adverse Effect Level, OECD, Organisation for Economic Co-operation and Development, TFT, 5-trifluorothymidine, USP, United States Pharmacopeia, WE-I, Williams E medium-Incomplete, Calcium L-methylfolate, L-5-MTHF-Ca, Toxicity, Genotoxicity, Developmental toxicity

## Abstract

Calcium L-methylfolate (L-5-MTHF-Ca; CAS Number 151533-22-1) is a source of folate and an alternative to folic acid for use in human food and food supplements. The safety of L-5-MTHF-Ca was evaluated by testing for genotoxicity, subchronic and prenatal developmental toxicity. In *in vitro* assays L-5-MTHF-Ca was not mutagenic and did not induce other chromosomal events. Additionally, L-5-MTHF-Ca was not genotoxic in the *in vivo* micronucleus test nor did it induce DNA damage in rat liver cells. In a subchronic toxicity study, rats administered up to 400 mg/kg bw/day of L-5-MTHF-Ca *via* oral gavage for 13 weeks had no treatment-related mortalities, and no treatment-related effects were identified on behaviour, body weight, food consumption, ophthalmology, haematology, or organ weights. No treatment-related macroscopic or histopathological findings were observed. Calcium and sodium levels increased with increasing dosage, however the slight increases were within historical control ranges and reversible after the recovery period. L-5-MTHF-Ca is neither teratogenic nor embryotoxic. Based on the results of the *in vitro* and *in vivo* studies, the safe use of L-5-MTHF-Ca as an ingredient in foods is supported. The no observed adverse effect level was the highest dose in the subchronic toxicity study, *i.e.* 400 mg/kg bw/day for male and female rats.

## Highlights

• The toxicity of calcium L-methylfolate was studied.
• No toxic, mutagenic, teratogenic nor embryogenic effects were observed.
• The NOAEL for calcium L-methylfolate was 400 mg/kg bw/day.

## 1 Introduction

Folate is a generic term referring to a family of water-soluble B vitamins that play an essential role in the synthesis of DNA and RNA, methionine regeneration, and in reactions required for normal cell metabolism and regulation [1]. Maintaining an adequate folate status is particularly important during periods of rapid cell proliferation and tissue growth such as gestation and infancy. Folate deficiency has been associated with increased risk for neural tube defects, certain chronic diseases and cancer [[2], [3], [4]]. Folate is naturally present in a wide range of foods with dark green leafy vegetables, legumes, peanuts, wheat bran and wheat germ serving as particularly rich sources [5]. The predominant form of naturally present folate in food is L-5-methyltetrahydrofolate (L-5-MTHF). Following consumption of folate-containing food, several monoglutamate forms of folate cross the intestinal lining and are converted into L-5-MTHF which is normally the only form of folate to enter the human circulation [6]. The folate vitamer L-5-MTHF is the main storage form in the human body and it is also the predominant form of folate in breast milk [7,8].

Many foods naturally rich in folates are consumed in amounts that are insufficient to meet the recommended dietary folate intake levels, therefore folic acid food fortification programs have been implemented and daily dietary supplementation with folic acid or the calcium salt of L-5-MTHF are recommended to improve folate status and prevent adverse effects related to folate deficiency [[9], [10], [11]]. Ingested L-5-MTHF-Ca dissociates readily and completely in the aqueous environment into Ca^2+^ and L-5-MTHF ions which pass through the intestinal mucosal cells [6]. Dietary supplementation with L-5-MTHF-Ca has several advantages over folic acid. As the predominant naturally present form of folate in food, serum and breast milk, ingested L-5-MTHF requires only glutamation during absorption and then it can directly enter the circulation, whereas folic acid does not occur naturally in foods in significant amounts and before being used must first be converted to L-5-MTHF in several enzymatic steps [6]. The physiological capacity of the enzymes involved are exceeded when folic acid is ingested at concentrations >400 μg/d resulting in exposure to unmetabolized folic acid through plasma and breast milk which may have negative effects [7,12,13]. The activity of the enzymes involved in conversion of folic acid to L-5-MTHF are influenced by several common polymorphisms in humans which reduce the capacity of the enzymes thereby reducing the bioavailability of folic acid to affected individuals [14,15].

Increasingly L-5-MTHF-Ca supplementation is being investigated for potential treatment of patients suffering from various human diseases or conditions such as Alzheimer’s disease [16], depression [17] and other psychiatric and neurologic conditions [18]. Supplementation with L-5-MTHF-Ca may have cognitive benefits particularly for subjects with elevated levels of serum homocysteine or low folate levels.

To evaluate the general safety of L-5-MTHF-Ca a battery of comprehensive toxicity tests were performed including tests for genotoxicity and mutagenicity, a prenatal developmental toxicity study in rats and a 13-week subchronic toxicity study in rats. Potential genotoxicity was investigated using a bacterial reverse mutation assay, a mouse lymphoma assay, an *in vivo* unscheduled DNA synthesis test and an *in vivo* micronucleus test.

## 2 Materials and methods

### 2.1 Study locations and guidelines

The battery of toxicity tests were performed in compliance with the Organisation for Economic Co-operation and Development (OECD) Principles of Good Laboratory Practice (GLP) regulations and in accordance with OECD Guidelines for Testing of Chemicals. The bacterial reverse mutation test (Ames test), *in vitro* mammalian cell gene mutation test, *in vivo* micronucleus test and prenatal developmental toxicity study were conducted at the Institute of Toxicology of Merck KGaA in Darmstadt, Germany according to OECD Guidelines 471, 476, 474, and 414 respectively. The *in vivo* and *in vitro* unscheduled DNA synthesis (UDS) test was performed at Covance Laboratories, Harrogate, England according to OECD Guideline 486. The 13-week oral toxicity study was performed at RCC Ltd, Itingen, Switzerland in accordance with OECD Guideline 408.

### 2.2 Test item

The test article, L-5-MTHF-Ca (CAS Number 151533-22-1), was supplied by Merck KGaA (Schaffhausen, Switzerland). Batch numbers of the test article used in the studies were: ESF-118 in the Ames test; LMCA-7077 in the micronucleus test, the mammalian cell gene mutation test and the 13-week subchronic oral toxicity study; LMCA-7290 in the unscheduled DNA synthesis and the prenatal developmental toxicity study. The purity of all test item batches ranged from 96.9 to 99.9%. The test article complies with specifications for calcium L-5-methyltetrahydrofolate listed in the US Pharmacopeia (USP) Dietary supplement monograph (USP 38) and in the Joint FAO/WHO Expert Committee on Food Additives (JECFA) Combined Compendium of Food Additives. The stability of room temperature aqueous solutions of L-5-MTHF-Ca for at least 2 h was confirmed by High Performance Liquid Chromatography (HPLC) analysis by RCC Ltd (Itingen, Switzerland).

### 2.3 Animals and organisms

For the bacterial reverse mutation assays *S. typhimurium* strains TA98, TA100, TA1535 and TA1537 were obtained from B.N. Ames at the University of Berkeley (California, USA), *S. typhimurium* strain TA102 was obtained from B. Diener at the University of Mainz (Germany), and *E. coli* strain WP2 uvrA (pkM101) was distributed from the National Collections of Industrial & Marine Bacteria Ltd (Aberdeen, Scotland) and obtained from H. Träger, Knoll AG (Ludwigshafen, Germany).

L5178Y TK^(+/−)^ mouse lymphoma cells for the *in vitro* mammalian gene mutation test were originally obtained frozen in liquid N_2_ from Dr. W. Muster at Hoffmann-La Roche (Basel, Switzerland).

For the *in vivo* mammalian erythrocyte micronucleus test male Wistar rats (HsdCpb:WU) were obtained from Harlan Winkelmann GmbH (Borchen, Germany). After at least 7 days acclimatization, animals in good health were randomly assigned to test groups. Average age of rats at dosing was 8- to 10-week-old and mean body weights (±SD) of the treatment groups ranged from 268 ± 6.03 to 280 ± 9.92 g. Rats were housed individually in Makrolon type 3 cages with softwood chippings and maintained at 23–24 °C, 57–60% humidity and with a light phase from 6 a.m. to 6 p.m. Altromin standard diet TPF N 1324 (10 mm pellets, poor in nitrosamines) and tap water were freely provided.

Male Han Wistar (Crl:WI (Glx/BRL/Han) BR) rats from Charles River Ltd (Margate, UK) were obtained for the unscheduled DNA synthesis test. Rats were acclimated for at least 5 days then randomly assigned to groups of four with individual group weight <5% different from the mean. Animals were at 19–25 °C, 40–70% relative humidity with a 12-h light/dark cycle and free access to water and food (Special Diets Services Ltd, RM1.(E).SQC).

For the 13-week subchronic oral toxicity, 6-week-old male and female Hanlbm:Wistar (SPF) rats obtained from RCC Ltd (Füllinsdorf, Switzerland) without any visible signs of illness were acclimatised for 1 week then randomly assigned to four groups, two with 15 males and 15 females (Control and high-dose groups) and two with 10 males and 10 females (low- and mid-dose groups). Body weight means per group and sex were comparable. At acclimatisation, body weights ranged from 118 to 162 g (mean 140 g) for the males and 112 to 142 g (mean 128 g) for females. Animals were housed in groups of five in Makrolon cages with standardised softwood bedding in an environment maintained at 22 ± 3 °C, 40–70% relative humidity with a 12-h light/dark cycle. Animals had free access to water and Kliba 3433 pelleted standard rat maintenance diet (Provimi Kliba AG, Kaiseraugst, Switzerland).

For the prenatal developmental toxicity study, virgin female Wistar rats (HsdCpb:WU) obtained from Harlan Winkelmann (Borchen, Germany) were acclimatised for 6 days before start of mating. The 12- to 13-week-old females (initial body weight range 181–231 g, mean 200 g) were left overnight in groups of 4 with a stock stud. The following day vaginal smears were taken to check for presence of semen. The day when sperm was detected was defined as gestation day 0. Females were then randomly assigned to one of four treatment groups (25/group). Animals were housed in Makrolon type III cages with softwood granulate bedding in an environment maintained at 22.5–25 °C, 39–49% relative humidity with a 12-h light/dark cycle. Animals had free access to water and Kliba 3433 pelleted standard rat maintenance diet (Provimi Kliba AG, Kaiseraugst, Switzerland).

### 2.4 Experimental design

#### 2.4.1 Bacterial reverse mutation assay

The Ames test [[19], [20], [21], [22]]) was conducted using two independent plate incorporation experiments with *Salmonella typhimurium* strains TA98, TA100, TA102, TA1535, TA1537 and *Escherichia coli* WP2 uvrA (pkM101). Both experiments were performed with and without metabolic activation by liver S9-mix prepared in-house from Aroclor 1254-pretreated male Wistar rats (S9-mix). L-5-MTHF-Ca was tested at concentrations of 5.00, 15.8, 50.0, 158, 1580 and 5000 μg/plate in experiment 1, and 50.0, 158, 158, 1580 and 5000 μg/plate in experiment 2. Double distilled water was the solvent (and negative control) and test concentrations were prepared by serial dilution. Positive controls in the absence of S9-mix were: daunomycin, N-ethyl-N'-nitro-N-nitrosoguanidine, 9-aminoacridine and cumene hydroperoxide; and in the presence of S9-mix: 2-aminoanthracene and benzo[a]pyrene (BaP). All treatments were plated in triplicate, except that 6 plates were used for the negative control. Revertant colonies were scored using an electronic colony counter (Artek Systems Corp., Farmingdale, NY) or manually. Data were not analyzed statistically.

#### 2.4.2 *In vitro* mammalian gene mutation test

Cell survival and mutation frequency of L5178Y TK^(+/−)^ mouse lymphoma cells exposed to L-5-MTHF-Ca were determined using a fluctuation protocol developed by Cole et al. (1983) [23]. Batches of L5178Y TK^(+/−)^ cells stored in liquid N_2_ were purged of TK^(+/−)^ mutants and checked for spontaneous mutant frequency and absence of mycoplasma. For each experiment rapidly thawed cells were grown in RPMI 1640-medium with GlutaMAX 1 supplement (Gibco, Grand Island, NY) containing 10% heat-inactivated horse serum and 1% penicillin/streptomycin (designated RPMI-10). Subcultures were established when cells were growing well. S9-mix was prepared in house in male Wistar rats according to Ames et al. (1975) [22] except that Dulbecco’s phosphate buffered saline (PBS) containing 20 mM HEPES pH 7.4 was used instead of KCl solution.

To prepare cell cultures, at least 10^7^ L5178Y TK^(+/−)^ cells in RPMI 1640-medium with GlutaMAX 1 supplement with 5% heat-inactivated horse serum and 1% penicillin/streptomycin (designated RPMI-5) were exposed to 50–5000 μg/ml of L-5-MTHF-Ca while shaking at 37 °C. Exposure time was 3 h with S9-mix and 24 h without S9-mix in series 1. In series 2 and 3 in the absence of S9-mix exposure times were 3 and 24 h, respectively. Solvent and positive controls (4-nitroquinoline N-oxide or BaP in dimethyl sulfoxide (DMSO)) were included for each assay and each treatment was performed in duplicate, except positive controls were in single cultures only. After 3 or 24 h exposure, cells were centrifuged, washed, resuspended in 10 ml RPMI-10 and cell concentration adjusted to 2 × 10^5^/ml. Cultures were diluted to be plated for survival or transferred to flasks for growth through the expression period.

To test for survival an aliquot of each culture was diluted to 8 cells/ml with RPMI-20 (RPMI 1640-medium with GlutaMAX 1 supplemented with 20% heat-inactivated horse serum and 1% penicillin/streptomycin) and 0.2 ml/well was placed into microtiter plates and incubated until scorable (day 6–10). Wells with viable clones were stained with 3-[4,5-dimethylthiazole-2-yl]-2,5-diphenylbromide (MTT), identified by eye and counted.

For the mutagenicity assay, cultures were maintained at cell densities below 1 × 10^6^ cells/ml for 2 or 3 days for expression of the TK^−^ mutation. Based on observed recovery and growth, cultures were selected to be plated for viability and mutagenicity. To test for viability, cell concentrations in the selected cultures were adjusted to 1 × 10^4^/ml and aliquots diluted to 8 cells/ml. Samples were dispensed on microtiter plates and incubated as described by Cole et al. (1983) [23] until scorable (d 7–10). Wells were stained with MTT and those containing viable clones were identified by eye and counted. Resistance to 5-trifluorothymidine (TFT) was determined by adding TFT (final concentration, 3 μg/ml) to cultures selected at the end of the expression period adjusted to cell densities of 1 × 10^4^/ml. TFT-treated cultures were dispensed into microtiter plates. Mutant selection started 2 d after start of the treatment in series 1 and 2 and 3 d after start in the series 3. Plates were incubated as described by Cole et al. (1983) [23] until scorable (d 10–14). For controls and positive test material effects mutation frequency was determined separately for small and large colonies in addition to the total mutation frequency. Data were not analyzed statistically.

#### 2.4.3 *In vivo* mammalian erythrocyte micronucleus test

L-5-MTHF-Ca was given once by oral gavage to male rats at the limit dose of 2000 mg/kg body weight. This dose was selected based upon a preliminary range-finding experiment showing no toxic effects on three male or three female rats at this maximum dose. Negative control group rats received the solvent, *i.e.* an oral dose of 10 ml 0.25% aqueous Methocel K4M premium/kg bw. Positive control animals were treated with an oral dose of 16.5 mg cyclophosphamide/kg bw. Five animals per dose group were used. Rats were observed for clinical symptoms after treatment and weighed daily.

Bone marrow smears were prepared from one femur per animal according to a modified version of the method described by Schmid [24]. For the L-5-MTHF-Ca dose groups, preparation took place at 24 and 48 h after administration of the test material. For the positive and negative control groups, the preparation time was 24 h after treatment start. After euthanisation by CO_2_, epiphyses were cut off the femur and bone marrow cells flushed out with foetal calf serum. The suspension was filtered according to Romagna (1988) and centrifuged for 5 min at 150 x g [25]. The sediment was resuspended in foetal calf serum and smears prepared. Slides were dried for 3 h then stained according to a modified Giemsa-staining method described by Gollapudi and Kamra (1979) using Giemsa's solution and Weise buffer solution and mounted in Entellan [26].

For microscopic investigation, one slide from each animal preparation was coded. The number of polychromatic erythrocytes with micronuclei per 2000 polychromatic erythrocytes per animal was determined. The quotient of normochromatic to polychromatic erythrocytes was calculated based on the analysis of 1000 erythrocytes per animal. The micronucleated normochromatic erythrocytes were registered when scoring the polychromatic erythrocytes. The number of micronucleated normochromatic erythrocytes 1000 erythrocytes was then calculated with the aid of the quotient.

#### 2.4.4 Unscheduled DNA synthesis test

L-5-MTHF-Ca was tested for its ability to induce unscheduled DNA synthesis in hepatocytes of male Han Wistar (Crl:WI (Glx/BRL/Han) BR) rats using an *in vivo*/*in vitro* procedure [27]. L-5-MTHF-Ca was dissolved in the vehicle, 0.25% w/v aqueous Methocel K4M, and administered once *via* oral gavage at doses of 2000 or 800 mg/kg to groups of four rats. Dose volume was 10 ml/kg bw. Rats were sacrificed 12–14 h after dosing in experiment 1 and 2–4 h after dosing in experiment 2. Negative control groups for each exposure time received the vehicle at the same volume. Positive control groups were dosed (10 ml/kg bw) with of 10 mg/kg dimethylnitrosamine dissolved in purified water (2–4 h experiment) or 75 mg/kg 2-acetamidofluorene suspended in corn oil (12–14 h experiment).

Hepatocytes were prepared after perfusion of livers from three animals per dose group with buffer containing calcium and collagenase. Cultures were diluted to 1.5 × 10^5^ viable cells. Each well of a 6-well multiplate was filled with 3 ml of the hepatocyte suspension, and then plates were incubated at 37 ± 1 °C in 5% CO_2_ in air (v/v) for at least 90 min.

Cells were radiolabelled and prepared for autoradiography as described by Kennelly et al., 1993 using Williams E medium-Incomplete (WE-I) containing 10 μCi/ml [^3^H] thymidine [27]. After 4 h incubation, cells were washed three times with WE-I containing 0.25 mM unlabelled thymidine and cultures were incubated overnight in the medium. Slides were coated in Ilford K2 liquid emulsion, gelled over ice for 10 min, incubated in a light-tight box at room temperature for 90 min., and then stored in the refrigerator for 14 d in sealed light-tight boxes with desiccant. Slides were developed in Kodak D19 developer and fixed using Ilford Hypam fixer. Cell nuclei and cytoplasm were stained with Meyers Haemalum/eosin Y and slides were dehydrated in ethanol, cleared in xylene and mounted with coverslips. Cells were scored. Nuclear and cytoplasmic grain counts were recorded and net grains/nucleus (NNG) calculated. Per animal 100 cells were analysed, using 2 or 3 slides in each case where possible.

### 2.5 Subchronic oral toxicity study

#### 2.5.1 Study design and test material administration

Test article in double distilled water was prepared daily at the testing facility and administered to animals *via* daily oral gavage (10 ml/kg bw) at concentrations of 0 (Control), 25 (low-dose), 100 (mid-dose) or 400 (high-dose) mg/kg bw/day. Dose levels were selected based upon publicly available information on toxicological studies performed with the racemate (D/L-5-MTHF-Ca) [28]. A 26-week study in rats showed deviations in body weight and clinico-chemical parameters in rats given daily oral doses of 120 and 360 mg/kg bw/d. Stability, homogeneity and test article concentration in gavage formulations was verified by HPLC at RCC Ltd (Itingen, Switzerland) before treatment start and homogeneity and concentration were analysed monthly. After 13 weeks (91 d for males, 92 d for females) ten animals per sex and group were sacrificed. Extra animals in the control and high-dose groups were retained for an additional 4-week treatment-free recovery period before being sacrificed.

#### 2.5.2 Animal observations

Food consumption per cage and body weight (bw) per animal were measured weekly. Animals were observed twice daily for mortality and viability and once daily cageside for clinical signs. Detailed clinical observations in home cages, in a standard arena and in the hand were performed weekly. Forelimb and hind limb grip strength were measured in triplicate using a push-pull strain gauge (Mecmedin, AGF 25 N, Slinfold, UK) at weeks 13 and 17. Animals were placed with forepaws inside a triangular grasping ring and hind paws outside a triangular grasping ring. Using one hand the animals were held towards the base of the tail and steadily pulled away or towards the ring until the grip was broken. Locomotor activity was measured quantitatively in randomised animals for 60 min in 15-min intervals during week 13 with an Activity Monitor 1052 System (Benwick Electronic Equipment Design Manufacture, Benwick, UK). Ophthalmoscopic examination of both eyes was performed on all animals prior to begin of the study and on control and high-dose animals at weeks 13 and 17. A mydriatic solution was applied followed by examination using a Miroflex 2 Ophthalmoscope (Heine, Herrsching, Germany).

#### 2.5.3 Haematology and clinical biochemistry

Blood samples were drawn from all animals at 13 weeks (day 91/92) and from the recovery period animals after 17 weeks for haematology and plasma chemistry measurements. Samples were taken early in the working day from lightly anaesthetized rats after fasting for about 18 h. Samples were drawn from the retro-orbital plexus using a micro-haematocrit glass capillary tube. The anticoagulants EDTA-K2 (haematology), lithium heparin (methaemoglobin and clinical biochemistry samples), and sodium citrate (coagulation) were used during blood collection. Erythrocyte count, haemoglobin concentration, haematocrit, mean corpuscular volume, mean corpuscular haemoglobin, mean corpuscular haemoglobin concentration, platelet count, reticulocyte count, reticulocyte fluorescence ratios, nucleated erythrocytes, Heinz bodies, methaemoglobin, total leukocyte counts, differential leukocyte counts, red blood cell morphology, thromboplastin time and activated partial thromboplastin time were measured. Clinical biochemistry measurements included glucose, urea, creatinine, uric acid, total bilirubin, total cholesterol, triglycerides, phospholipids, aspartate aminotransferase, alanine aminotransferase, lactate dehydrogenase, creatine kinase, alkaline phosphatase, gamma-glutamyl transferase, total protein and concentration of Ca, P, Na, K, Cl.

#### 2.5.4 Urinalysis

Urine was collected into specimen vials using a metabolism cage during the 18 h fasting period prior to blood sampling after weeks 13 and 17. Analyses included evaluation of volume, specific gravity, osmolality, colour, appearance, pH, protein, glucose, ketone, bilirubin, blood, nitrite, urobilinogen, sediment, red blood cells, and crystals.

#### 2.5.5 Histopathology

After 13 or 17 weeks of treatment, animals were euthanised by exsanguination following anaesthetisation *via* intraperitoneal injection of sodium pentobarbitone. Animals were weighed and necropsied. Weights were recorded for brain, heart, liver, thyroids and parathyroids, thymus, kidneys, adrenals, uterus, spleen, testes, epididymides and ovaries. Relative organ to terminal body weight ratios and organ to brain ratios were calculated. All macroscopic abnormalities were recorded. Samples of the weighed organs listed above and the following organs and tissues were collected from all animals at necropsy and fixed in neutral phosphate buffered 4% formaldehyde solution (except where indicated): aorta, bone (sternum, femur including joint), bone marrow (femur), cecum, colon, duodenum, esophagus, exorbital lacrimal glands, eyes with optic nerve (fixed in Davidson's solution), Harderian gland (fixed in Davidson's solution), ileum, with Peyer's patches, jejunum with Peyer's patches, larynx, lacrimal gland exorbital, lungs (infused with formalin at necropsy), lymph nodes (mesenteric, mandibular), mammary gland area, nasal cavity (turbinates), pancreas, pituitary gland, prostate gland, rectum, salivary glands (mandibular, sublingual), sciatic nerve, seminal vesicles, skeletal muscle, skin, spinal cord (cervical, midthoracic, lumbar), stomach, tongue, trachea, urinary bladder (infused with formalin at necropsy), vagina and any gross lesions. Organ and tissue samples were processed, embedded, cut to *ca.* 2–4 μm thickness and stained with haematoxylin and eosin. Histological examinations were performed on preserved organs and tissues from all control and high dose animals, and all gross lesions noted in any of the test groups at the time of terminal sacrifice.

### 2.6 Prenatal developmental toxicity study

#### 2.6.1 Study design and test material administration

The test article in 0.25% aqueous hydroxypropyl methylcellulose was prepared daily at the testing facility under subdued lighting and kept in brown glass bottles at room temperature and stirred continuously during administration. Formulation concentrations were verified once during the first week of treatment at the Institute of Toxicology, Merck KGaA using a validated spectrophotometric method. Test article was administered to animals *via* daily oral gavage (10 ml/kg bw) on 15 consecutive days from gestation day (GD) 5 to 19 at concentrations of 0 (Control), 100 (low-dose), 300 (mid-dose) or 1000 (high-dose) mg/kg bw.

#### 2.6.2 Animal observations and examinations

Behaviour and appearance were checked twice daily. Body weight of female rats was measured on GD 0, and then daily from GD 5 to 20. Food consumption was measured on GD 5, 10, 15, 20. Water consumption was measured on GD 3, 6, 9, 12, 15, 18, 20.

On GD 20 all females were anaesthetised by inhalation, abdominal cavities were opened and ovaries and uteri removed. Gravid uteri were weighed. Bodies without uteri and foetuses were weighed and recorded as terminal body weights. The number of corpora lutea in both ovaries was determined and the number of live and dead foetuses and resorptions were determined. Resorptions were recorded as: complete (occurred shortly after implantation and detected after staining of uteri with ammonium sulphide), early (diameter ≤0.5 cm, no macroscopic differentiation between foetal and placental residues possible) or late (diameter usually >0.5 cm, clear macroscopic differentiation between foetal and placental residues possible). The total number of implantations was determined by ammonium sulphide staining. Females without implantation sites were recorded as being non-pregnant.

Foetuses were examined macroscopically for abnormal deviations and weighed. Sex was determined based on anogenital distance and inspection of gonads. In each treatment group all foetuses were numbered and those with uneven numbers of each litter were scheduled for transverse section. Foetuses were fixed in formalin and dissected transversely (method of Wilson, 1965) and inspected for organ malformations (modified Barrow and Taylor, 1969) [29,30]. Even numbered foetuses were eviscerated and examined for organ malformations. Eviscerated foetuses were fixed in alcohol and treated according to the double straining method of Whitaker and Dix (1979), *i.e.* the soft parts were cleared with KOH solution, skeletons were stained with Alizarin Red S, and the cartilage with Alcian Blue. Foetuses were then examined for skeletal abnormalities and malformations [31].

### 2.7 Statistical analyses

#### 2.7.1 *In vivo* mammalian erythrocyte micronucleus test

Statistical analyses were performed on the number of micronuclei-containing polychromatic erythrocytes per animal. Each treatment group was compared to the negative control. For comparisons, the exact Mann-Whitney-test (SPSS-System (Version 9.0) under Windows NT) was used against one-sided alternatives.

#### 2.7.2 Subchronic oral toxicity study

Statistical analyses were performed by RCC on body and organ weights, all ratios, and clinical chemistry parameters. Means and standard deviations were calculated for quantitative data, with male and female rats evaluated separately. Treatment groups were compared to control groups using a one-way analysis of variance (ANOVA). The Dunnett-test based on a pooled variance estimate was applied if the variables could be assumed to follow a normal distribution for the comparison of the treated groups and the control groups for each sex. The Steel-test was applied when the data could not be assumed to follow a normal distribution. Locomotor activity and grip strength data were analyzed using a Student’s T-Test. Fisher’s exact-test was applied to opthalmoscopy data and macroscopic findings.

#### 2.7.3 Developmental study

To compare parameters to the control the two sided Dunnett test was used for body weight, body weight gain, terminal body weight, foetus weight, and food and water consumption. The Fisher-Pitman Permutation test with adjustment according to Bonferroni-Holm under consideration of k≥2 treatment groups was used to statistically analyse corpora lutea implantations, live foetuses, percent life foetuses, percent resorptions, dead and malformed foetuses, and percent post implantation loss [32].

## 3 Results

### 3.1 Genotoxicity studies

#### 3.1.1 Bacterial reverse mutation assay

L-5-MTHF-Ca did not induce a clear or dose-dependent increase in the number of revertant colonies in any of the tester strains with- or without S9-metabolic activation in either of the two Ames test experiments (data not shown). To reach the highest concentrations, *i.e.* 500–5000 μg/plate, the material was plated as a suspension. In both experimental series there were no signs of cytotoxicity of L-5-MTHF-Ca to the bacteria (*i.e.* no reduction in number of spontaneous revertants and no clearing of the background lawn was observed). Negative control mutant frequencies were within acceptable ranges designated according to Levin et al. (1982) and Kier et al. (1986), and the historical controls of the laboratory (TA 98: 15–60; TA 100: 75–200; TA 102: 200–450; TA 1535: 3–37; TA 1537: 4–31; WP2: 50–200) (slight deviations in only one test series were accepted when all other parameters of that series were in the regular range) [33,34]. Positive control compounds induced a clear increase in the number of revertants over the negative control, thereby showing the expected reversion properties of all strains and good metabolic activation of the S9-mix. Results obtained for the negative and positive controls confirmed the validity of the assay. L-5-MTHF-Ca did not cause an increase in mean number of revertant colonies in any of the tester strains with or without metabolic activation and there were no dose-related increases in mutations with- or without S9-metabolic activation. Therefore, L-5-MTHF-Ca was considered to be non-mutagenic under the experimental conditions.

#### 3.1.2 *In vitro* mammalian gene mutation test

L-5-MTHF-Ca was found to be non-mutagenic in mouse lymphoma cells at concentrations of 50.0, 158, 500, 1580, 2810 and 5000 μg L-5-MTHF-Ca/ml. The maximum concentration was selected after a preliminary range-finding assay showed reduced cell relative survival at the highest concentration tested without a relevant change in pH and osmolarity. Mean mutant frequencies of negative controls met validity requirements of less than 2-fold the historical mean values (130 × 10^−6^ without S9, 146 × 10^−6^ with S9) in series 1 and series 2 (Table 1), but not in series 3 (2.2-times the historical mean). The positive control in series 1 without S9 and series 2, met the validity requirements of a mutation frequency at least 2-fold above the highest value of the historical negative controls (241 × 10^−6^) and at least a 4-fold increase in mutation frequency of the actual negative control. The positive control in series 1 with S9 gave a mutation frequency 2-fold above the highest value of the historical negative controls but only 2.5-fold higher than the actual negative control, thereby not meeting validity requirements. Validity requirements for the assay were only met by series 1 without S9 and series 2 experiments. Colony characterisation by colony size of the negative and positive controls and the highest concentrations of L-5-MTHF-Ca confirm that both the large and small colony mutants were adequately detected.

**Table 1:** Mutagenicity and cytotoxicity of L-5-MTHF-Ca in mouse lymphoma cells.

Series (Duration)	Test article	Dose (μg/ml)	S9	Precipitation^a^	%RS^b^	%RTG^c^		MF^d^		
								total	small	large
1 (3h)	Solvent	0	+	–	100	100		233	110	117
	L-5-MTHF-Ca	50	+	–	111	153		147	ND	ND
		158	+	PB	129	170		205	ND	ND
		500	+	PE	126	121		133	ND	ND
		1580	+	PE	149	162		130	ND	ND
		2810	+	PE	131	236		247	ND	ND
		5000	+	PE	128	123		299	ND	ND
	BaP^e^	2.00	+	–	133	115		446	177	246
		3.00	+	–	81	111		589	259	314
1 (24h)	Solvent	0	–	–	100	100		178	51	113
	L-5-MTHF-Ca	50	–	–	96	101		215	ND	ND
		158	–	–	94	88		209	ND	ND
		500	–	PE	98	91		174	ND	ND
		1580	–	PE	101	40		197	ND	ND
		2810	–	PE	103	13		403	139	234
		5000	–	PE	65	4	614		236	307
	NQO^f^	0.10	–	–	42	24	884		198	509
		0.20	–	–	30	15	1508		390	659
2 (3h)	Solvent	0	–	–	100	100	246		64	175
	L-5-MTHF-Ca	50	–	–	82	84	235		ND	ND
		158	–	PE	82	85	242		ND	ND
		500	–	PE	102	76	267		ND	ND
		1580	–	PE	99	50	282		ND	ND
		2810	–	PE	76	29	344		ND	ND
		5000	–	PE	43	14	450		140	346
	NQO^e^	0.10	–	–	58	44	754		221	472
		0.20	–	–	34	25	984		253	848
3 (24h)	Solvent	0	–	–	100	100	289		53	216
	L-5-MTHF-Ca	50	–	–	93	109	289		ND	ND
		158	–	PE	88	77	454		ND	ND
		500	–	PE	93	79	348		ND	ND
		1580	–	PE	152	53	327		ND	ND
		2810	–	PE	181	30	343		ND	ND
		5000	–	PE	47	0.2	865		159	733
	NQO^e^	0.10	–	–	106	50	881		124	629
		0.20	–	–	51	42	967		81	791

a Precipitation at the beginning (PB) or end (PE) of experiment.

b Relative Survival (mean of 2 plates) as a percentage of the negative control.

c Relative Total Growth (mean of 2 plates) as a percentage of the negative control.

d 5-TFT Mutant Frequency per 10^6^ viable cells (mean of 2 plates).

e Benzo[a]pyrene.

f 4-Nitroquinoline N-oxide; + S9 present; - S9 absent; ND = not determined.

In the different experimental series precipitation of the test material occurred at concentrations ≥158 or ≥500 μg/ml and relative total growth values were very low for concentrations of L-5-MTHF-Ca above 1580 μg/ml. In series 1 without S9, mean mutation frequencies at 2810 and 5000 μg/ml L-5-MTHF-Ca were between 2- and 4-fold the mean actual negative control, thereby showing a weak effect on mutation frequency in this experiment. Mutation frequency in series 2 was less than 2-fold the actual negative control and L-5-MTHF-Ca was determined to have no effect on the mutation frequency. According to the evaluation criteria, test materials showing a weak effect in one series and no effect in another series are assessed as negative or non-mutagenic and it can be concluded that L-5-MTHF-Ca did not induce mutagenic effects at the TK locus in mouse lymphoma cells.

#### 3.1.3 *In vivo* mammalian erythrocyte micronucleus test

L-5-MTHF-Ca did not induce micronucleus formation in the immature erythrocytes of rats (data not shown). No clinical symptoms were observed in any of the animals receiving 2000 mg/kg bw L-5-MTHF-Ca and there was no treatment-related decrease in body weight. Mean number of micronuclei-containing cells per 1000 polychromatic erythrocytes for negative controls (0.50–2.00) were within the range of historical controls of the laboratory for male rats (0.5–2.2). The positive control resulted in an increase in polychromatic erythrocytes with micronuclei (*p* < 0.01) and values (10.0–20.5) were within the range of historical controls of the laboratory (8.0–22.0). L-5-MTHF-Ca did not induce a statistically significant or biologically relevant increase in the number of polychromatic erythrocytes with micronuclei compared to the negative control and the number of normochromatic cells with micronuclei, calculated per 1000 normochromatic erythrocytes, was not increased. There was a significant (*p* < 0.05) increase in the quotient of normochromatic to polychromatic erythrocytes in the L-5-MTHF-Ca 48 h exposure time group, 2.50 compared to 1.32 of the negative control group, a possible result of depressed erythroblast proliferation as a result of cytotoxicity, and a valuable indication of bone-marrow exposure by the test agent. L-5-MTHF-Ca did not induce micronucleus formation in the immature erythrocytes of rats in the study, therefore, L-5-MTHF-Ca was considered to be non-genotoxic under the conditions of the study.

#### 3.1.4 Unscheduled DNA synthesis test

L-5-MTHF-Ca did not induce DNA damage that could be repaired by unscheduled DNA synthesis in liver cells of rats given oral gavage doses of 800 and 2000 mg/kg bw, neither at 2–4 h, nor at 12–14 h sampling time (Table 2). No clinical signs of toxicity or weight loss were observed in any main study animal. Negative (vehicle) controls gave a group mean NNG value below zero with only 0.3-0.7% of cells in repair; both parameters were within the range of historical controls. Positive controls induced increases in NNG to more than 17 and more than 50% of cells were in repair. The assay was therefore accepted as valid. Treatment with 800 or 2000 mg/kg L-5-MTHF-Ca did not produce a group mean NNG value greater than -1.9 nor were more than 2.7% cells found in repair at either dose. These data confirm that L-5-MTHF-Ca does not have genotoxic effects on rat livers.

**Table 2:** Unscheduled DNA synthesis in rat liver cells following L-5-MTHF-Ca exposure.

Dose (mg/kg)	Nuclear grain count	Cytoplasmic grain count	Net nuclear grain count (NNG)	Cells in repair (NNG ≥ 5) %
	Mean ± SD			
	**2-4 h Experiment**			
Negative control	2.84 ± 0.47	4.27 ± 0.21	−1.58 ± 0.41	0.3 ± 0.6
800	2.99 ± 0.35	5.07 ± 1.03	−2.08 ± 0.73	0.7 ± 1.2
2000	3.06 ± 0.77	4.97 ± 0.66	−1.92 ± 0.17	0.3 ± 0.6
Positive control	21.06 ± 4.03	3.38 ± 0.50	17.68 ± 3.55	98.7 ± 1.5
	**12-14 h Experiment**			
Negative control	6.68 ± 1.73	9.61 ± 4.05	−2.93 ± 2.33	0.7 ± 0.6
800	6.62 ± 1.92	9.50 ± 3.09	−2.88 ± 1.29	1.0 ± 1.7
2000	6.73 ± 0.94	9.12 ± 1.61	−2.39 ± 1.88	2.7 ± 1.5
Positive control	52.38 ± 3.94	9.14 ± 1.43	43.24 ± 4.16	100.0 ± 0.0

*N* = 3 animals per group; 300 cells per group counted, *i.e.* 100 cells per animal.

#### 3.1.5 Subchronic oral toxicity study

There were no mortalities related to the administration of L-5-MTHF-Ca*.* A single female in the high-dose treatment group died following a gavage error which was confirmed at necropsy. All other animals survived until scheduled necropsy.

Analysis of L-5-MTHF-Ca formulations in distilled water confirmed the stability and homogeneity of the gavage test solutions at room temperature over a period of 2 h.

Clinical observations were limited to bright faeces observed in the mid- and high-dose L-5-MTHF-Ca groups. These were attributed to the beige-yellow colour of the test article and considered toxicologically irrelevant. Daily and weekly clinical and functional observations revealed isolated incidences reported equally in control and test item-treated animals and not related to L-5-MTHF-Ca administration. Locomotor activity in 15-min time intervals was different for some of the treatment groups compared to the control, but differences did not occur in both sexes, did not show a clear dose relationship and total activity over the 60-min interval was not different between experimental groups, therefore, differences in locomotor activity were considered incidental. Lower mean hind limb grip strength in the mid-dose (*p* < 0.01) and high-dose (*p* < 0.05) L-5-MTHF-Ca females at Week 13 (587g and 622g, respectively verses 700g in the control) was considered incidental as it was not accompanied by differences in forelimb strength, a clear dose relationship was lacking, and no other signs of muscular weakness or motor neurotoxicity in either sex were observed.

In females administered L-5-MTHF-Ca, mean weekly body weights, body weight gains and feed consumption were comparable to those in the control during both the main treatment and the recovery periods. In males, mean body weight of the mid-dose group was numerically lower compared to the other groups at the study start and significantly lower (*p* < 0.05) than the control on days 22, 29, 36 and 43. At all other time points during the main study and recovery period, the mean body weight of mid-dose males was not significantly different from the control. Furthermore mean body weight was not reduced in the high-dose group and there were no differences in body weight gain or feed consumption in the main or recovery periods between any treatment groups. Therefore, the transient reduction in the mean body weight of the mid-dose was considered incidental.

Ophthalmological examination (Week 13) identified two incidences of corneal opacity in the control (one per sex) and three in high-dose males, in addition four incidences of persistent pupillary membrane in the control (one male, three females) and the high-dose treatment group (three males, one female) were observed. Both optical abnormalities have been reported to occur spontaneously and in high frequencies in Wistar and Han rats [35,36]. Ocular examination results were not statistically significant for either sex during the pre-test and at the end of the treatment period; thus the isolated incidences were not attributed to L-5-MTHF-Ca administration.

There were no statistically significant changes in haematology or coagulation parameters in males at the end of the treatment (Table 3). In high-dose females at Week 13 the relative segmented neutrophil count increased and the relative lymphocyte count decreased (*p* < 0.05; data not shown). The differences in the relative counts of the two haematological parameters stems from the numerical but not significant increase in absolute neutrophil counts in high-dose females (Table 3). The parameters were not significantly different after the recovery period (not shown). As total blood cell counts were unchanged, there were no accompanying histopathological effects or a dose-response effect, all haematology and coagulation means were within the laboratory’s historical control range observed in the age and strain of rat used, and there was no evidence of infection or inflammation, the differences were considered to be not toxicologically relevant.

**Table 3:** Haematology and coagulation parameters in rats following 13 weeks of treatment with L-5-MTHF-Ca.

Parameter (units)	Sex	Control 0 mg/kg bw/d	Low-dose 25 mg/kg bw/d	Mid-dose 100 mg/kg bw/d	High-dose 400 mg/kg bw/d
***Haematology***					
RBC (10^6^/μl)	M	9.05 ± 0.40	9.26 ± 0.42	9.17 ± 0.45	9.12 ± 0.36
	F	8.26 ± 0.26	8.35 ± 0.26	8.20 ± 0.37	8.25 ± 0.30
HB (mmol/l)	M	10.04 ± 4.87	10.16 ± 0.28	10.29 ± 0.33	10.26 ± 0.27
	F	9.57 ± 0.41	9.47 ± 0.34	9.46 ± 0.44	9.55 ± 0.23
HCT (l/l)	M	0.47 ± 0.01	0.47 ± 0.01	0.47 ± 0.01	0.47 ± 0.01
	F	0.45 ± 0.02	0.45 ± 0.02	0.45 ± 0.02	0.45 ± 0.01
MCV (fl)	M	51.6 ± 2.1	50.9 ± 2.4	51.8 ± 2.8	52.0 ± 1.5
	F	54.7 ± 1.35	53.1 ± 2.7	54.7 ± 1.1	54.8 ± 1.3
MCH (fmol)	M	1.11 ± 0.05	1.10 ± 0.05	1.13 ± 0.06	1.12 ± 0.03
	F	1.16 ± 0.03	1.14 ± 0.03	1.16 ± 0.03	1.16 ± 0.03
MCHC (mmol/l)	M	21.5 ± 0.2	21.6 ± 0.2	21.7 ± 0.3	21.6 ± 0.3
	F	21.2 ± 0.3	21.4 ± 0.9	21.1 ± 0.3	21.2 ± 0.3
Platelet count (10^3^/μl)	M	806 ± 102	811 ± 134	887 ± 135	879 ± 80
	F	837 ± 78	884 ± 74	929 ± 104	914 ± 130
WBC (10^3^/μl)	M	6.6 ± 1.7	6.8 ± 2.1	5.9 ± 1.4	6.3 ± 1.2
	F	4.1 ± 1.3	4.0 ± 1.1	3.8 ± 0.8	4.5 ± 1.1
ANC (10^3^/μl)	M	1.27 ± 0.48	1.48 ± 0.52	1.20 ± 0.41	1.04 ± 0.32
	F	0.63 ± 0.45	0.64 ± 0.22	0.55 ± 0.17	0.87 ± 0.29
ALC (10^3^/μl)	M	5.18 ± 1.55	5.26 ± 1.69	4.62 ± 1.32	5.18 ± 1.10
	F	3.43 ± 1.01	3.23 ± 1.02	3.22 ± 0.81	3.51 ± 0.83
AMC (10^3^/μl)	M	0.01 ± 0.04	0.04 ± 0.04	0.03 ± 0.05	0.01 ± 0.02
	F	0.01 ± 0.02	0.01 ± 0.03	0.00 ± 0.00	0.01 ± 0.02
AEC (10^3^/μl)	M	0.09 ± 0.09	0.06 ± 0.06	0.07 ± 0.06	0.08 ± 0.06
	F	0.05 ± 0.04	0.06 ± 0.05	0.06 ± 0.05	0.07 ± 0.06
ABC (10^3^/μl)	M	0.00 ± 0.00	0.00 ± 0.00	0.00 ± 0.00	0.00 ± 0.00
	F	0.00 ± 0.00	0.00 ± 0.00	0.00 ± 0.00	0.00 ± 0.00
ARC (10^3^/μl)	M	212.8 ± 27.9	226.1 ± 23.0	213.7 ± 28.5	217.0 ± 26.9
	F	225.1 ± 28.7	227.4 ± 30.0	227.5 ± 28.5	236.7 ± 29.6
***Coagulation***					
PT (sec)	M	12.7 ± 0.5	12.4 ± 0.4	12.7 ± 0.5	12.7 ± 0.8
	F	12.1 ± 0.3	12.0 ± 0.3	12.3 ± 0.3	12.1 ± 0.5
APTT (sec)	M	25.0 ± 2.2	24.3 ± 4.3	24.0 ± 1.7	24.8 ± 2.3
	F	22.1 ± 2.0	21.6 ± 1.4	23.0 ± 1.0	22.8 ± 1.3

ABC, absolute basophil count; AEC, absolute eosinophil count; ALC, absolute lymphocyte count; AMC, absolute monocyte count; ANC, absolute segmented neutrophil count; APTT, activated partial thromboplastin time; ARC, absolute reticulocyte count; HB, haemoglobin; HCT, haematocrit; MCH, mean corpuscular haemoglobin; MCHC, mean corpuscular haemoglobin concentration; MCV, mean corpuscular volume; PT, prothrombin time; RBC, erythrocyte count; WBC, total white blood cell count; Mean ± SD shown; *N* = 15 Group 1 and Group 4 males; *N* = 10 Group 2 and 3, males and females; *N* = 14 Group 1 and Group 4 females.

Statistically significant changes in clinical chemistry results from Week 13 included lower uric acid and triglycerides (*p* < 0.05) in mid-dose males and lower aspartate aminotransferase (AST) (*p* < 0.01) and alanine aminotransferase (ALT) (*p* < 0.05) in mid-dose females compared to controls (Table 4). These differences were not dose-related and were within the laboratory’s historical range for the sex, species and age of rat used and were, therefore, judged not to be toxicologically relevant. Cholesterol and phospholipid levels were higher in high-dose females compared to the controls (*p* < 0.05), whereas levels in males were numerically higher but did not reach statistical significance. Values were well within the laboratory’s historical range, and findings were not accompanied by any histopathological changes or other indicators of hepatocellular or cholestatic liver injury and effects were reversible, *i.e.* differences did not persist through the recovery period (Table 5). Consequently, differences were considered to be not toxicologically relevant. Aspartate aminotransferase levels were lower in high-dose males (*p* < 0.05) but were within the historical range, were not accompanied by any other indications of toxicological relevance and were note different after the recovery period, therefore were considered not toxicologically relevant. Mean lactate dehydrogenase (LDH) levels in all male L-5-MTHF-Ca-treatment groups were numerically lower than the control group, reaching statistical significance in the high-dose group. The measured mean LDH level for the control group was outside the laboratory’s historical range (1.09 to 5.72 μkat/l) and the value for the mid-dose group was slightly outside the historical range, whereas mean values for low- and high-dose groups were within the historical range. After the recovery phase LDH levels of the control and high-dose group were not significantly different and were both within the historical range (Table 5). Similarly, creatine kinase levels were numerically lower than those in the control group in all L-5-MTHF-Ca-treated males, reaching significance in the low- and high dose groups. Again, the control group was outside the laboratory’s historical range (1.31 to 5.36 μkat/l) while mean values in the test article-treated groups were within the historical range, and after the recovery phase creatine kinase levels of the control and high-dose group (2.66 and 2.12, respectively) were not significantly different and were both within the historical range. Female rats in the high dose group had significantly higher creatine kinase levels after the recovery period although treatment groups weren’t significantly different following the main study. As all measured parameters indicated healthy liver, kidneys and heart, it was concluded that the LDH and creatine kinase measurements in the control group were spuriously high, and differences were not toxicologically relevant in relation to the test article. In both sexes calcium levels increased with L-5-MTHF-Ca concentration reaching significance in the high-dose group and sodium levels were significantly higher in mid- and high-dose animals. The differences did not persist during the recovery period and all values were within the historical range of the laboratory, therefore, the increase was seen as reversible and not adverse.

**Table 4:** Clinical chemistry values following 13 weeks of treatment with L-5-MTHF-Ca.

Parameter (units)	Sex	Control 0 mg/kg bw/d	Low-dose 25 mg/kg bw/d	Mid-dose 100 mg/kg bw/d	High-dose 400 mg/kg bw/d
Glucose (mmol/l)	M	5.88 ± 0.74	6.00 ± 0.54	6.08 ± 0.52	5.59 ± 0.86
	F	5.18 ± 0.79	5.27 ± 0.55	5.34 ± 0.83	5.03 ± 0.66
Urea (mmol/l)	M	5.62 ± 0.57	5.86 ± 0.96	5.52 ± 0.56	5.52 ± 0.87
	F	7.22 ± 0.77	6.79 ± 0.81	7.06 ± 0.78	6.86 ± 0.88
Creatine (μmol/l)	M	49.8 ± 2.0	49.4 ± 1.9	49.9 ± 2.7	49.3 ± 1.8
	F	53.3 ± 2.9	52.6 ± 3.3	52.8 ± 2.6	54.2 ± 2.9
Uric acid (μmol/l)	M	32.7 ± 17.3	20.3 ± 6.1	17.8 ± 7.9*	33.0 ± 13.7
	F	34.5 ± 13.6	27.6 ± 14.2	22.9 ± 6.4	43.2 ± 17.9
Bilirubin, total (μmol/l)	M	1.53 ± 0.33	1.47 ± 0.37	1.72 ± 0.36	1.57 ± 0.42
	F	1.98 ± 0.44	1.91 ± 0.56	1.71 ± 0.59	2.15 ± 0.79
CHOL, total (mmol/l)	M	1.72 ± 0.33	1.79 ± 0.33	1.78 ± 0.40	1.94 ± 0.41
	F	1.30 ± 0.30	1.57 ± 0.24	1.43 ± 0.43	1.69 ± 0.44*
Triglycerides (mmol/l)	M	0.43 ± 0.13	0.35 ± 0.11	0.31 ± 0.06*	0.35 ± 0.08
	F	0.29 ± 0.04	0.30 ± 0.07	0.31 ± 0.05	0.30 ± 0.06
Phospholipids (mmol/l)	M	1.44 ± 0.21	1.49 ± 0.23	1.47 ± 0.24	1.56 ± 0.21
	F	1.44 ± 0.26	1.64 ± 0.17	1.53 ± 0.35	1.76 ± 0.34*
AST (μkat/l (37 °C))	M	1.43 ± 0.32	1.28 ± 0.15	1.33 ± 0.17	1.21 ± 0.08*
	F	1.50 ± 0.46	1.36 ± 0.25	1.11 ± 0.09**	1.30 ± 0.19
ALT (μkat/l (37 °C))	M	0.55 ± 0.10	0.51 ± 0.07	0.58 ± 0.13	0.51 ± 0.07
	F	0.52 ± 0.16	0.49 ± 0.07	0.41 ± 0.03*	0.46 ± 0.08
LDH (μkat/l (37 °C))	M	7.09 ± 2.47	5.26 ± 1.91	5.98 ± 1.40	5.13 ± 1.34*
	F	4.64 ± 2.22	4.78 ± 1.87	3.49 ± 1.15	5.28 ± 1.55
Creatine kinase (μkat/l (37 °C))	M	7.73 ± 5.95	4.20 ± 1.75*	4.36 ± 1.60	3.55 ± 0.79**
	F	4.23 ± 3.41	4.44 ± 1.79	2.83 ± 1.60	3.74 ± 1.73
ALP (μkat/l (37 °C))	M	2.56 ± 0.54	2.55 ± 0.24	2.61 ± 0.45	2.43 ± 0.25
	F	1.29 ± 0.31	1.25 ± 0.27	1.13 ± 0.18	1.08 ± 0.22
G-GT (nkat/l (37 °C))	M	17.59 ± 5.27	18.24 ± 5.93	19.30 ± 9.31	20.64 ± 4.86
	F	11.88 ± 6.70	11.85 ± 6.90	12.05 ± 4.88	12.18 ± 5.68
Calcium (mmol/l)	M	2.69 ± 0.04	2.72 ± 0.05	2.71 ± 0.06	2.74 ± 0.06*
	F	2.77 ± 0.09	2.78 ± 0.07	2.80 ± 0.08	2.85 ± 0.06*
Phosphorus (mmol/l)	M	1.64 ± 0.11	1.75 ± 0.09	1.75 ± 0.15	1.76 ± 0.17
	F	1.37 ± 0.24	1.51 ± 0.31	1.40 ± 0.26	1.38 ± 0.23
Sodium (mmol/l)	M	145.9 ± 0.9	146.6 ± 1.3	147.2 ± 1.0*	147.1 ± 1.3*
	F	146.5 ± 0.7	147.3 ± 1.2	148.5 ± 1.1**	148.0 ± 1.1**
Potassium (mmol/l)	M	4.09 ± 0.21	4.07 ± 0.17	3.94 ± 0.30	4.07 ± 0.32
	F	3.57 ± 0.29	3.62 ± 0.26	3.54 ± 0.28	3.49 ± 0.36
Chloride (mmol/l)	M	106.9 ± 1.0	107.1 ± 0.9	107.5 ± 1.0	107.1 ± 1.4
	F	110.6 ± 1.3	109.6 ± 1.8	110.3 ± 1.1	109.3 ± 1.5
Protein, total (g/l)	M	67.0 ± 2.3	66.8 ± 1.6	66.4 ± 2.6	67.6 ± 1.5
	F	70.5 ± 5.0	70.4 ± 3.1	71.0 ± 2.9	73.0 ± 3.2

Values are given as mean ± SD. *N* = 15 males and 15 females in Group 1, 10 males and 10 females in Group 2 and Group 3, 15 males and 14 females in Group 4. ALP, alkaline phosphatase; ALT, alanine aminotransferase; AST, aspartate aminotransferase; CHOL, cholesterol; GGT, gamma-glutamyl transferase; LDH, lactate dehydrogenase.

* *p*<0.05, compared to Group 1 (control).

** *p*<0.01 compared to Group 1 (control).

**Table 5:** Clinical chemistry values after the additional 4-week recovery period.

Parameter (units)	Sex	Control 0 mg/kg bw/d	High-dose 400 mg/kg bw/d
Glucose (mmol/l)	M	4.92 ± 0.70	5.19 ± 0.25
	F	5.43 ± 0.62	5.22 ± 0.81
Urea (mmol/l)	M	5.40 ± 0.30	5.26 ± 0.56
	F	7.27 ± 1.32	7.50 ± 0.35
Creatine (μmol/l)	M	50.0 ± 2.5	48.8 ± 1.4
	F	55.7 ± 1.5	59.7 ± 5.1
Uric acid (μmol/l)	M	46.7 ± 18.3	39.3 ± 14.3
	F	33.6 ± 26.0	52.8 ± 17.9
Bilirubin, total (μmol/l)	M	1.14 ± 0.20	1.30 ± 0.27
	F	1.34 ± 0.49	1.65 ± 0.70
CHOL, total (mmol/l)	M	1.58 ± 0.28	2.02 ± 0.54
	F	1.43 ± 0.31	1.36 ± 0.17
Triglycerides (mmol/l)	M	0.37 ± 0.09	0.37 ± 0.07
	F	0.37 ± 0.06	0.34 ± 0.05
Phospholipids (mmol/l)	M	1.32 ± 0.17	1.62 ± 0.27
	F	1.56 ± 0.18	1.47 ± 0.15
AST (μkat/l (37 °C))	M	1.30 ± 0.21	1.13 ± 0.05
	F	1.47 ± 0.30	1.31 ± 0.17
ALT (μkat/l (37 °C))	M	0.55 ± 0.08	0.54 ± 0.07
	F	0.56 ± 0.17	0.38 ± 0.04
LDH (μkat/l (37 °C))	M	3.20 ± 1.34	2.08 ± 0.61
	F	3.33 ± 1.17	4.85 ± 2.38
Creatine kinase (μkat/l (37 °C))	M	2.66 ± 0.22	2.12 ± 0.70
	F	3.28 ± 2.59	8.19 ± 3.27*
ALP (μkat/l (37 °C))	M	2.43 ± 0.38	2.41 ± 0.34
	F	1.14 ± 0.37	1.13 ± 0.08
G-GT (nkat/l (37 °C))	M	17.27 ± 4.73	12.14 ± 3.95
	F	17.50 ± 4.28	14.29 ± 3.57
Calcium (mmol/l)	M	2.71 ± 0.05	2.70 ± 0.05
	F	2.68 ± 0.09	2.66 ± 0.10
Phosphorus (mmol/l)	M	1.67 ± 0.12	1.71 ± 0.17
	F	1.14 ± 0.23	1.07 ± 0.27
Sodium (mmol/l)	M	147.3 ± 0.4	147.3 ± 0.4
	F	146.8 ± 1.2	146.9 ± 1.3
Potassium (mmol/l)	M	4.13 ± 0.38	4.02 ± 0.31
	F	3.49 ± 0.36	3.57 ± 0.45
Chloride (mmol/l)	M	104.5 ± 0.5	104.4 ± 0.6
	F	105.5 ± 1.7	105.4 ± 1.6
Protein, total (g/l)	M	68.0 ± 3.1	67.9 ± 1.7
	F	70.7 ± 7.0	69.5 ± 2.6

Values are given as mean ± SD. *N* = 5 males and 5 females in Group 1, 5 males and 4 females in Group 4. ALP, alkaline phosphatase; ALT, alanine aminotransferase; AST, aspartate aminotransferase; CHOL, cholesterol; GGT, gamma-glutamyl transferase; LDH, lactate dehydrogenase.

* *p*<0.05, compared to Group 1 (control).

Urinalysis parameters of L-5-MTHF-Ca-treated female rats were not different from the control group. In males, specific gravity and osmolality were significantly reduced in some test article-treated groups accompanied by significantly higher pH in high-dose males (Table 6). The significant differences did not persist through the recovery period (not shown). All values remained within the 95% tolerance limits of the historical control data, were transient, and no morphological changes were observed, therefore, these findings were considered to be non-adverse. There were no differences between treatment groups for scores of protein, glucose, ketone, bilirubin, blood, nitrite, urobilinogen, sediment, red blood cells, and crystals in either sex (not shown).

**Table 6:** Urinalysis parameters following 13 weeks of treatment with L-5-MTHF-Ca.

Parameter (units)	Sex	Control 0 mg/kg bw/d	Low-dose 25 mg/kg bw/d	Mid-dose 100 mg/kg bw/d	High-dose 400 mg/kg bw/d
Urine volume (mL)	M	6.9 ± 1.5	7.5 ± 1.8	6.8 ± 1.7	7.5 ± 1.3
	F	5.9 ± 1.8	6.9 ± 1.7	5.8 ± 1.1	5.7 ± 1.3
Specific gravity	M	1.037 ± 0.005	1.030 ± 0.005**	1.031 ± 0.004**	1.029 ± 0.005**
	F	1.034 ± 0.005	1.030 ± 0.004	1.031 ± 0.003	1.031 ± 0.004
Osmolality (mmol/kg)	M	1364 ± 221	1107 ± 175**	1209 ± 194	1091 ± 173**
	F	1259 ± 237	1142 ± 172	1235 ± 156	1255 ± 187
pH	M	6.3 ± 0.3	6.5 ± 0.3	6.6 ± 0.3	6.8 ± 0.3+
	F	6.0 ± 0.5	6.3 ± 0.4	6.1 ± 0.2	5.9 ± 0.3

Values are given as mean ± SD. *N* = 15 males and 15 females in Group 1, 10 males and 10 females in Group 2 and Group 3, 15 males and 14 females in Group 4.

** *p*<0.01 compared to Group 1 (control) Dunnett-test based on pooled variance.

+ *p*<0.01 compared to Group 1 (control) Steel-test.

Absolute organ weights and organ-to-body/brain weight ratios did not support any toxicologically relevant effects related to the test article in male (Table 7) or female (Table 8) rats. At the end of the treatment period the only difference in organ weights in male rats was observed in the mid-dose group which had significantly (*p* < 0.05) lower mean absolute epididymes weight than the control group. Significant differences were not observed when calculated relative to body or brain weigh and no dose-related effect was seen. After the recovery period, the epididymus in high-dose males was numerically heavier than the control, reaching significance when relative weight compared to the brain weight was calculated (data not shown). In females none of the treatment group absolute organ weights were different from the control group. Calculated relative to body weight, the ovaries were significantly smaller in low-dose females and kidneys were larger in mid-dose females. When calculated relative to brain weight, treatment high-dose females had larger liver and kidneys compared to the control. The differences in liver and kidney relative weight did not persist throughout the recovery period. Taken together, there were no differences in organ or relative organ weights attributed to treatment with the test article.

**Table 7:** Organ weights of male rats following 13 weeks of treatment with L-5-MTHF-Ca.

Parameter	Control 0 mg/kg bw/d	Low-dose 25 mg/kg bw/d	Mid-dose 100 mg/kg bw/d	High-dose 400 mg/kg bw/d
*Absolute organ weight (g)*				
Body weight	365.245 ± 33.959	370.547 ± 26.189	343.075 ± 18.388	359.141 ± 31.914
Brain	2.09 ± 0.12	2.05 ± 0.09	2.02 ± 0.08	2.06 ± 0.08
Heart	1.102 ± 0.171	1.083 ± 0.089	1.053 ± 0.089	1.068 ± 0.096
Thyroids	0.028 ± 0.005	0.026 ± 0.002	0.024 ± 0.008	0.026 ± 0.004
Liver	9.28 ± 1.17	9.09 ± 1.07	8.44 ± 0.61	8.91 ± 0.98
Thymus	0.28 ± 0.06	0.32 ± 0.07	0.29 ± 0.08	0.32 ± 0.06
Kidneys	2.11 ± 0.23	2.12 ± 0.23	2.03 ± 0.20	2.03 ± 0.24
Adrenals	0.061 ± 0.011	0.058 ± 0.007	0.058 ± 0.008	0.058 ± 0.007
Spleen	0.753 ± 0.086	0.699 ± 0.075	0.678 ± 0.117	0.748 ± 0.119
Testes	3.77 ± 0.22	3.68 ± 0.26	3.62 ± 0.25	3.63 ± 0.32
Epididymides	1.452 ± 0.130	1.450 ± 0.133	1.295 ± 0.162*	1.355 ± 0.108
*Organ-to-body weight ratio (%)*				
Brain	0.57 ± 0.04	0.55 ± 0.04	0.59 ± 0.04	0.58 ± 0.06
Heart	0.301 ± 0.024	0.293 ± 0.027	0.307 ± 0.024	0.298 ± 0.024
Thyroids	0.008 ± 0.001	0.007 ± 0.001	0.007 ± 0.002	0.007 ± 0.001
Liver	2.54 ± 0.18	2.45 ± 0.17	2.46 ± 0.19	2.48 ± 0.15
Thymus	0.08 ± 0.01	0.09 ± 0.02	0.08 ± 0.02	0.09 ± 0.02
Kidneys	0.58 ± 0.04	0.57 ± 0.03	0.59 ± 0.06	0.57 ± 0.05
Adrenals	0.017 ± 0.003	0.016 ± 0.002	0.017 ± 0.002	0.016 ± 0.001
Spleen	0.207 ± 0.021	0.188 ± 0.014	0.198 ± 0.033	0.209 ± 0.031
Testes	1.04 ± 0.10	1.00 ± 0.08	1.06 ± 0.09	1.02 ± 0.09
Epididymides	0.400 ± 0.040	0.391 ± 0.025	0.378 ± 0.043	0.379 ± 0.036
*Organ-to-brain weight ratio (%)*				
Heart	52.582 ± 6.113	52.824 ± 3.944	52.002 ± 3.559	51.890 ± 4.898
Thyroids	1.329 ± 0.201	1.249 ± 0.102	1.195 ± 0.403	1.263 ± 0.243
Liver	444.24 ± 48.13	442.96 ± 44.48	417.04 ± 24.84	433.50 ± 53.14
Thymus	13.57 ± 2.33	15.73 ± 3.86	14.28 ± 3.77	15.46 ± 2.63
Kidneys	100.74 ± 7.59	103.32 ± 9.11	100.55 ± 9.47	98.57 ± 11.22
Adrenals	2.908 ± 0.417	2.813 ± 0.343	2.848 ± 0.382	2.804 ± 0.346
Spleen	36.017 ± 3.274	34.124 ± 3.679	33.451 ± 5.165	36.225 ± 5.160
Testes	180.64 ± 10.72	179.74 ± 9.77	178.63 ± 9.55	176.45 ± 14.99
Epididymides	69.644 ± 6.690	70.715 ± 5.213	63.990 ± 7.431	65.741 ± 4.533

Values are given as mean ± SD (*N* = 10 in each group).

* *p*<0.05 compared to Group 1 (control).

**Table 8:** Organ weights of female rats following 13 weeks of treatment with L-5-MTHF-Ca.

Parameter	Control 0 mg/kg bw/d	Low-dose 25 mg/kg bw/d	Mid-dose 100 mg/kg bw/d	High-dose 400 mg/kg bw/d
*Absolute organ weight (g)*				
Body weight	228.571 ± 12.595	234.124 ± 10.012	227.903 ± 15.869	236.188 ± 18.716
Brain	1.97 ± 0.08	1.93 ± 0.07	1.96 ± 0.07	1.93 ± 0.07
Heart	0.816 ± 0.040	0.828 ± 0.064	0.815 ± 0.070	0.851 ± 0.078
Thyroids	0.021 ± 0.004	0.021 ± 0.003	0.020 ± 0.004	0.022 ± 0.005
Liver	5.85 ± 0.41	6.24 ± 0.37	6.07 ± 0.75	6.45 ± 0.83
Thymus	0.30 ± 0.06	0.28 ± 0.05	0.32 ± 0.07	0.32 ± 0.07
Kidneys	1.39 ± 0.13	1.48 ± 0.11	1.50 ± 0.15	1.49 ± 0.10
Adrenals	0.077 ± 0.016	0.075 ± 0.012	0.080 ± 0.019	0.080 ± 0.014
Spleen	0.601 ± 0.091	0.585 ± 0.053	0.588 ± 0.082	0.542 ± 0.203
Ovaries	0.121 ± 0.024	0.100 ± 0.016	0.111 ± 0.020	0.120 ± 0.024
Uterus	1.058 ± 0.291	0.976 ± 0.185	0.916 ± 0.300	1.071 ± 0.271
*Organ-to-body weight ratio (%)*				
Brain	0.87 ± 0.05	0.83 ± 0.05	0.86 ± 0.07	0.82 ± 0.06
Heart	0.358 ± 0.029	0.354 ± 0.030	0.358 ± 0.020	0.361 ± 0.025
Thyroids	0.009 ± 0.002	0.009 ± 0.002	0.009 ± 0.002	0.010 ± 0.002
Liver	2.56 ± 0.17	2.67 ± 0.17	2.67 ± 0.32	2.73 ± 0.21
Thymus	0.13 ± 0.02	0.12 ± 0.02	0.14 ± 0.03	0.14 ± 0.03
Kidneys	0.61 ± 0.04	0.63 ± 0.04	0.66 ± 0.06*	0.63 ± 0.04
Adrenals	0.034 ± 0.007	0.032 ± 0.006	0.035 ± 0.007	0.034 ± 0.005
Spleen	0.263 ± 0.035	0.250 ± 0.025	0.258 ± 0.036	0.227 ± 0.080
Ovaries	0.053 ± 0.009	0.043 ± 0.006*	0.049 ± 0.008	0.051 ± 0.011
Uterus	0.462 ± 0.123	0.417 ± 0.080	0.404 ± 0.140	0.452 ± 0.098
*Organ-to-brain weight ratio (%)*				
Heart	41.390 ± 2.884	42.989 ± 4.255	41.572 ± 3.779	44.171 ± 3.723
Thyroids	1.057 ± 0.167	1.107 ± 0.187	1.023 ± 0.179	1.166 ± 0.242
Liver	296.52 ± 17.49	323.42 ± 18.07	309.88 ± 40.68	334.83 ± 40.92*
Thymus	15.08 ± 3.25	14.38 ± 2.06	16.36 ± 3.56	16.81 ± 3.59
Kidneys	70.37 ± 5.29	76.57 ± 5.67	76.66 ± 7.11	77.30 ± 4.90*
Adrenals	3.882 ± 0.812	3.884 ± 0.739	4.088 ± 0.904	4.151 ± 0.712
Spleen	30.456 ± 4.593	30.331 ± 2.744	30.053 ± 4.849	28.076 ± 10.477
Ovaries	6.105 ± 1.168	5.217 ± 0.851	5.624 ± 0.940	6.248 ± 1.399
Uterus	53.584 ± 14.749	50.802 ± 10.749	46.906 ± 16.400	55.795 ± 15.024

Values are given as mean ± SD (*N* = 10 in each group).

* *p*<0.05 compared to Group 1 (control).

Histological sections of the tissues identified in the methods section for histopathology were examined by light microscopy and findings were graded in severity using a five point system of minimal, slight, moderate, marked or severe. Occasional findings were recorded as present or as unilateral or bilateral. Findings in tissues are reported in Table 9. Tissues identified for histopathology and not included in the table did not have findings. Two high-dose females had moderate or slight centriacinar (periportal) intrahepatic brown pigmentation and increased Kupffer cell pigment. This finding was occasionally observed in control rats at the laboratory and therefore its presence in these animals was considered to be fortuitous rather that a result of treatment with the test article. There were no treatment-related macroscopic or microscopic findings related to exposure to the test substance.

**Table 9:** Microscopic findings in rats following 13 weeks of treatment with L-5-MTHF-Ca.

Parameter	Control 0 mg/kg bw/d	Low-dose 25 mg/kg bw/d	Mid-dose 100 mg/kg bw/d	High-dose 400 mg/kg bw/d
***MALES***				
EPIDIDYMIDES – Focal mononuclear cell infiltration - Min	0	–	–	1
EYES - Periorbital inflammation - Min	1	–	–	0
EYES - Bilateral retinal atrophy - Mod	0	–	–	1
HEART – Focal myocarditis	3 (2 M in. 1 Sl)	–	–	1 (Min)
JEJUNUM - Mineralisation in Peyer’s patch - P	0	–	–	1
KIDNEYS – Focus (i) of basophilic (regenerating) tubules – Min	4	1	0	3
Mineralisation – pelvis and papilla -Sl	1	0	0	1
Unilateral hydronephrosis	2 (Min)	1 (Min)	1 (Sl)	3 (Min)
Bilateral hydronephrosis - Min	0	0	0	1
LIVER - Microfoci of inflammation	8 (Min)	–	0	8 (7 M in. 1 Sl)
Lobar necrosis - Mar	0	–	1	0
LUNG - Focus (i) of alveolar macrophages - Min	1	1	–	0
Focal pneumonitis - Min	0	0	–	1
Perivascular mononuclear cell infiltration - Min	0	0	–	1
LYMPH NODE, MESENTERIC – Sinusoidal erythrocytes - P	0	–	–	1
PANCREAS – Focal acinar atrophy – Min	0	–	–	1
PITUITARY GLAND - Cyst(s) – P	4	–	–	3
PROSTATE GLAND – Focal inflammatory cell infiltration – Sl	3	–	–	1
RECTUM – Nematodiasis – P	1	–	–	0
SEMINAL VESICLES - Congestion - P	1	–	–	0
SPLEEN - Extramedullary haematopoiesis - Min	2	–	–	0
***FEMALES***				
COLON - Nematodiasis – P	1	–	–	0
HEART – Focal myocarditis - Min	1	–	–	0
KIDNEYS – Focus (i) of basophilic (regenerating) tubules – Min	1	–	–	2
Mineralisation – pelvis and papilla–Sl	0	–	–	1
Mineralisation –inner and outer medullary stripes	9 (1 M in. 6 Sl, 2 Mod)	–	–	10 (1 M in. 8 Sl, 1 Mod)
Unilateral hydronephrosis - Min	1	–	–	1
LIVER - Microfoci of inflammation	7 (6 M in. 1 Sl)	–	–	5 (Min)
Centriacinar intrahepatic brown pigment	0	–	–	2 (1 Sl, 1 Mod)
Increased kupffer cell pigment	0	–	–	2 (1 Sl, 1 Mod)
Centriacinar fat vacuolation – Min	3	–	–	3
Basophilic (tigroid) focus (i) - Min	1	–	–	0
LYMPH NODE, MESENTERIC – Sinusoidal erythrocytes - P	0	–	–	1
Subdermal oedema – P	0	–	–	1
PANCREAS – Focal acinar atrophy – Min	2	–	–	1
RECTUM – Nematodiasis – P	1	–	–	0
SPLEEN - Extramedullary haematopoiesis	2 (Min)	–	–	4 (3 M in. 1 Sl)
Haemosiderin deposition – Sl	0	–	–	1
Mineralisation – Mod	0	–	–	1
UTERUS - Cyclical dilation and oedema – P	3	1	–	4
VAGINA – Dioestrous morphology – P	3	–	–	1
Proestrous morphology - P	3	–	–	4
Oestrous morphology – P	2	–	–	3
Metoestrous morphology - P	2	–	–	2

Preserved organs and tissues from all 10 control and 10 high dose animals and all gross lesions noted in any test groups at time of sacrifice were examined. P = present, Min = minimal, Sl = slight, Mod = moderate, Mar = marked, - = tissue not examined.

#### 3.1.6 Prenatal developmental toxicity study

There were no maternal deaths during the study. Of the 25 rats per group, 24 were pregnant in the control group, 23 in the low-dose group, 22 in the mid-dose group and 22 in the high-dose group on GD 20. There were two reported incidences of hair loss in the high-dose group and one in each of the other groups. One incidence of skin lesion was observed in the mid-dose group and one incidence of discharge from the nose in the high-dose group. Observed incidences were considered not treatment related.

None of the treatment groups differed from the control in respect to body weight, body weight gain or food consumption. Water consumption in the low- and mid-dose groups was comparable to the control. In the high-dose group water consumption increased slightly but was statistically significant between GD 15 and GD 20. This was considered to be non-adverse.

There were no abnormal findings in dams sacrificed on GD 20. Gravid uterus weight, number of corpora lutea and number of implantations in the treated groups were not significantly different from the control group. There were no treatment related effects on resorptions, number of foetuses, mean foetus weight or sex distribution. No differences in type and frequency of skeletal variations, soft tissue malformations or ossification status were observed in the treated groups compared to the control (Table 10). In addition, all evaluated parameters were in the normal range of the historical control.

**Table 10:** Investigations and findings in foetuses.

Findings	Control 0 mg/kg bw/d	Low-dose 100 mg/kg bw/d	Mid-dose 300 mg/kg bw/d	High-dose 1000 mg/kg bw/d
***External examination***				
Microphthalmia unilateral	–	–	–	1
Exencephalia	–	–	–	1
Micrognathia	–	–	–	1
Haematoma (ear, hind leg, or face)	1	1	–	1
***Evisceration***				
Fetuses with findings	–	–	–	–
**Skeleton stain**				
Asymmetric sternebrae	2	5	1	4
Fused sternebrae	1	–	–	1
Frontal, parietal, interparietal, and supraoccipital bone not ossified	–	–	–	1
***Transverse sections***				
Microphthalmia unilateral	1	–	–	–
Anophthalmia	–	1	–	–
Slight dilation of renal pelvis	5	6	2	2
Cystic dilation (fore brain)	1	–	–	–
Hydrocephalus	–	1	–	–
**Sum of fetuses affected/per fetuses investigated**	11/275	13/254	3/238	9/262
**Sum of litters affected/per litters investigated**	8/24	8/23	3/22	7/22

– = no incidences.

## 4 Discussion

In the bacterial reverse mutation assay (Ames test) L-5-MTHF-Ca did not cause gene mutations by base pair changes or frameshifts in the genome of the tester strains. L-5-MTHF-Ca also did not induce gene mutations or other chromosomal events in the mammalian cell gene mutation assay in mouse lymphoma cells. In the original study report of the mammalian cell gene mutation assay, the different experimental series were mistakenly not assessed for compliance with the validity requirements defined in the protocol and the conclusion was reached that L-5-MTHF-Ca was weakly mutagenic in the test system in the absence of S9-mix and under conditions where strong cytotoxic effects occurred. The original study report was submitted to the European Food Safety Authority (EFSA) as part of their assessment of the safety of L-5-MTHF-Ca for use in foods [37]. EFSA reviewed the original study report and agreed that in series 1 without S9 at the two highest test article concentrations the relative total growth was reduced to 13 and 4% of controls, respectively, and the mutation frequency was weakly increased. EFSA noted that according to the current evaluation criteria no reliable estimate of mutation frequency could be made at survival levels of less than 10%. The assay was performed according to OECD Guideline 476, which has since been replaced by OECD 490. The OECD 490 Guideline takes into consideration that excessive cytotoxicity can cause false positive responses. The study results have been reassessed in this publication, and taking into consideration the original validity requirements, the conclusion that L-5-MTHF-Ca was not mutagenic in the mammalian cell gene mutation assay was reached. L-5-MTHF-Ca also did not induce structural or numerical chromosomal damage in the immature erythrocytes of the rat in the *in vivo* micronucleus test. The non-genotoxic nature of L-5-MTHF-Ca was further confirmed in the unscheduled DNA synthesis assay in which L-5-MTHF-Ca did not induce DNA damage in rat liver cells to be repaired by unscheduled DNA synthesis. Under the conditions of these studies, L-5-MTHF-Ca was considered to be non-mutagenic with respect to gene mutations, structural and numerical chromosome aberrations, and did not cause genotoxic effects in the liver.

In the prenatal developmental toxicity study L-5-MTHF-Ca was well-tolerated by maternal animals and foetuses at doses up to 1000 mg/kg bw/d. Reproductive performance was not affected by the treatment. All pregnant females had litters with viable foetuses. None of the examined parameters (percent absorptions/litter, average number of live foetuses/litter, average foetal body weight/litter, and sex ratio/litter) was influenced by the treatment. Necropsy of the maternal rats did not reveal gross changes that could be attributed to the treatment. Examination of the foetuses for external, visceral and skeletal malformations and anomalies did not reveal any foetotoxic, embryotoxic or teratogenic effects of L-5-MTHF-Ca*.* Therefore, the No Observed Adverse Effect Level (NOAEL) for L-5-MTHF-Ca was considered to be the highest tested dose, 1000 mg/kg bw/d, for both maternal and developmental toxicity.

In the subchronic oral toxicity study, administration of L-5-MTHF-Ca *via* gavage at doses up to 400 mg/kg bw/d was well tolerated and had no negative effects on clinical observations, behaviour, ophthalmological or neurological parameters in male and female rats. All animals survived until scheduled necropsy except one female in the high-dose group which died after a gavage error. After the 13-weeks of test article administration, mean body weights, body weight gains and feed consumption were comparable to those in the control group. The functional observational battery and locomotor activity tests did not reveal any changes in response to the treatment. There were no treatment-related effects on haematological and coagulation parameters. Plasma analysis revealed significantly lower levels of aspartate amino-transferase, lactate dehydrogenase and creatine kinase in males but not in females of the high-dose group. These changes were small in magnitude the absolute and relative organ weights did not change in response to the treatment in both genders and histopathological examinations did not reveal any abnormalities that could be attributed to the treatment, therefore differences were considered to be non-adverse and reversible.

The results of the present study demonstrate an excellent safety profile for L-5-MTHF-Ca*.* There were no treatment-related effects noted at any dose level. Therefore, the NOAEL for L-5-MTHF-Ca was considered to be 400 mg/kg bw/d or at the highest tested dose in both male and female rats.

In addition to the herein described genotoxicity and toxicity studies in animals, the safety of L-5-MTHF-Ca has been demonstrated in human studies. The safety and tolerability of L-5-MTHF-Ca provided as daily doses up to 15 mg for 12 months was recently investigated in patients suffering from major depressive disorder [17]. The authors found no difference in reported adverse effects compared to the placebo group. Similarly, patients with hyperhomocysteinemia given daily doses of 15 mg L-5-MTHF-Ca for 2 months, or 15 mg L-5-MTHF-Ca on a cyclic program for 2 years (1 month therapy followed by 2 months withdrawal) did not report any side effects or particular complaints related to the L-5-MTHF-Ca supplementation [38,39].

Studies in healthy adults supplemented with daily doses of L-5-MTHF-Ca ranging from 400 to 500 μg for up to 24 weeks have also been reported [[40], [41], [42]]. The purpose of these studies was generally to compare the bioavailability of L-5-MTHF-Ca relative to folic acid and while no adverse effects related to L-5-MTHF-Ca were reported in the studies, the safety of L-5-MTHF-Ca to the subjects was not the main focus of the trials.

The results of the herein described toxicity studies support the safety of L-5-MTHF-Ca for the intended use in infant formula, food fortification and dietary supplements. These results are backed up by studies preformed in humans that, while not specifically designed to evaluate the safety of L-5-MTHF-Ca, do provide additional support for the safety of L-5-MTHF-Ca at doses up to 15 mg per day. L-5-MTHF-Ca is a safe alternative to folic acid as a source of folate and may be advantageous in particular for individuals with defects in the methylenetetrahydrofolate reductase enzyme who could have difficulty processing folic acid from supplements or fortified foods.

## Declaration of Competing Interest

The authors declare no conflict of interest.
